# Simulated effect of pneumococcal vaccination in the Netherlands on existing rules constructed in a non-vaccinated cohort predicting sequelae after bacterial meningitis

**DOI:** 10.1186/1471-2334-10-259

**Published:** 2010-09-03

**Authors:** Arno E Commandeur, Rogier CJ de Jonge, Irene Koomen, Lodewijk Spanjaard, A Marceline van Furth, Caroline B Terwee

**Affiliations:** 1 Department of Pediatrics and Infectious Diseases, VU University Medical Center, Amsterdam, The Netherlands; 2 Department of Neonatology, Emma Children's Hospital - Academic Medical Center, Amsterdam, The Netherlands; 3 Department of Pediatrics, Westfriesgasthuis, Hoorn, The Netherlands; 4 Department of Medical Microbiology, Netherlands Reference Laboratory for Bacterial Meningitis, Academic Medical Center, Amsterdam, The Netherlands; 5 Department of Epidemiology and Biostatistics and the EMGO Institute for Health and Care Research, VU University Medical Center, Amsterdam, The Netherlands

## Abstract

**Background:**

Previously two prediction rules identifying children at risk of hearing loss and academic or behavioral limitations after bacterial meningitis were developed. *Streptococcus pneumoniae *as causative pathogen was an important risk factor in both. Since 2006 Dutch children receive seven-valent conjugate vaccination against *S. pneumoniae*. The presumed effect of vaccination was simulated by excluding all children infected by *S. pneumoniae *with the serotypes included in the vaccine, from both previous collected cohorts (between 1990-1995).

**Methods:**

Children infected by one of the vaccine serotypes were excluded from both original cohorts (hearing loss: 70 of 628 children; academic or behavioral limitations: 26 of 182 children). All identified risk factors were included in multivariate logistic regression models. The discriminative ability of both new models was calculated.

**Results:**

The same risk factors as in the original models were significant. The discriminative ability of the original hearing loss model was 0.84 and of the new model 0.87. In the academic or behavioral limitations model it was 0.83 and 0.84 respectively.

**Conclusion:**

It can be assumed that the prediction rules will also be applicable on a vaccinated population. However, vaccination does not provide 100% coverage and evidence is available that serotype replacement will occur. The impact of vaccination on serotype replacement needs to be investigated, and the prediction rules must be validated externally.

## Background

About 5 per 100,000 children suffer from bacterial meningitis (BM) in the Netherlands per year [1]. BM is still a serious disease with a mortality rate of 5%, despite antibiotic treatment and advances in care of critically ill patients [2]. Approximately 15-20 percent of BM survivors develop severe acute or long term sequelae such as epilepsy, hydrocephalus or hearing loss. More subtle adverse outcomes such as cognitive or behavioral limitations are present in 20-30 percent of the children [3-7]. Some of these more subtle limitations are recognized in an early phase after the BM, but a major group has substantial delay in the detection of these problems. Early detection of sequelae and adequate treatment or follow up is of major importance since it may prevent worsening of symptoms and development of secondary problems.

We have previously developed two prediction rules to identify children at risk of developing hearing loss and academic or behavioral limitations after BM. These prediction rules consist of clinical variables which can be obtained during admission. The following variables were identified as risk factors for developing hearing loss: duration of symptoms prior to admission, absence of petechiae, cerebral spinal fluid (CSF) glucose level, *Streptococcus pneumoniae *as causative pathogen and ataxia. The prediction rule for academic or behavioral limitations included: gender, birth weight, educational level of the father, *S. pneumoniae *as causative pathogen, CSF leukocyte count, delay between admission and the start of antibiotics, the use of dexamethasone, seizures, and the duration of fever during admission. The rationale and design of the studies in which the prediction rules were developed have been extensively described in earlier manuscripts [5,6].

In both our prediction rules *S. pneumoniae *as causative pathogen is an important risk factor. Since June 2006 all Dutch newborn children receive a seven-valent conjugate vaccine (PCV-7) against this pathogen (serotypes 4,6B,9V,14,18C,19F,23F) on the age of 2,3,4 and 11 months.

Observations in other countries which introduced this vaccine earlier showed major decrease of BM and other infections caused by *S. pneumoniae *with the serotypes included in the vaccine. An increase in the incidence of the not included serotypes 19A, 6C, 22F and 15 was also observed [8-13]. As a result the population of children who might develop BM after vaccination is expected to be smaller and with different distribution of pneumococcal serotypes than the population on which the two prediction rules have been based. The aim of this study was to evaluate whether the existing prediction rules are also still applicable on a vaccinated population.

## Methods

This study consists of secondary data analyses of previously collected data, used to develop the prediction rules for developing hearing loss and academic or behavioral limitations. Approval for use of this data was obtained from the entitled researchers. The construction of these rules has been described extensively in the original publications [5,6]. In short, the design was as follows: all Dutch children born between January 1986 and December 1994 that recovered from BM caused by *Neisseria meningitidis*, *S. pneumoniae*, *Streptococcus agalactiae*, *Escherichia coli *or *Listeria monocytogenes *between January 1990 and December 1995 were eligible for the study. These children were selected from the files of the Netherlands Reference Laboratory for BM which collects strains and data from approximately 80% of all Dutch meningitis cases [1]. The diagnosis of BM was confirmed in CSF by culture of bacteria or by demonstrating bacterial antigens with latex agglutination. All patients with a complex onset of BM (defined as: meningitis secondary to immunodeficiency states, central nervous system surgery, cranial trauma or cerebrospinal fluid shunt infection, or relapsing meningitis), pre-existing cognitive or behavioral problems or severe handicaps were excluded [5,6].

Sixteen hundred and five children treated in 110 different Dutch hospitals met the criteria for inclusion. The pediatricians of these hospitals were asked to send the parents (or guardians) a standard letter requesting their participation. The parents who returned informed consent were sent screening questionnaires about health, learning and behavior, and were asked permission to study the medical records of their child.

Finally, 628 children were included in the cohort regarding hearing loss and 674 children in the cohort used for the development of the rule predicting academic or behavioral limitations [5,6].

Hearing loss was defined as a perceptive loss of >25 dB and was based on information from questionnaires and medical records. A total of 43 of the remaining 628 children were identified as having hearing loss. Information on the risk factors included in the prediction rule for hearing loss, was extracted from the medical records [5].

Academic or behavioral limitations were identified in two steps. The first step was based on parental perception on learning and behavior studied by two questionnaires. Based on the scores on the questionnaires the 674 children were divided into two groups: one with children with parents with complaints on learning and behavior (n = 134, 20%) and one with children with parents without these complaints (n = 540, 80%). For the second step a nested case-control study of 201 children was constructed. Therefore a random sample from both groups was drawn: 100 children from the group with and 101 from the group without suspected problems. The medical records of these 201 children were studied to identify the risk factors included in the prediction rule for academic or behavioral limitations. Exploration of these records sometimes provided new information on medical history, resulting in extra patients meeting exclusion criteria. Therefore another 19 of the 201 children were excluded, leaving a total of 182 children in the cohort (89 with suspected problems based on parental perception, 93 without). These 182 children were extensively tested on academic performance using a Academic Achievement Test (AAT) and on behavioral functioning with the Child Behavior Checklist (CBCL) [14]. Out of 182 children, 84 were identified as having academic or behavioral limitations, leaving 98 children without limitations [6].

**Figure 1:**
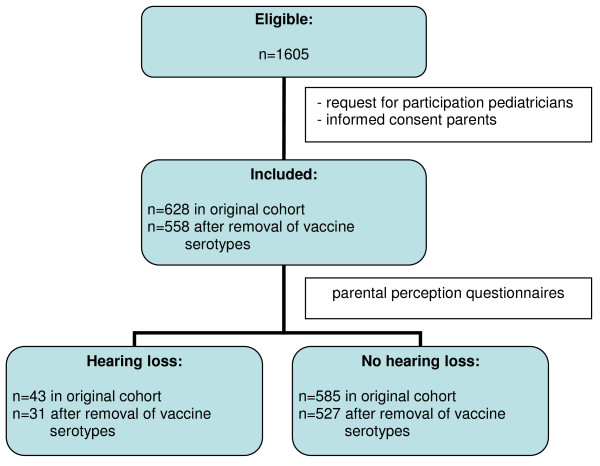
**patient flow chart for "hearing loss"**.

**Figure 2:**
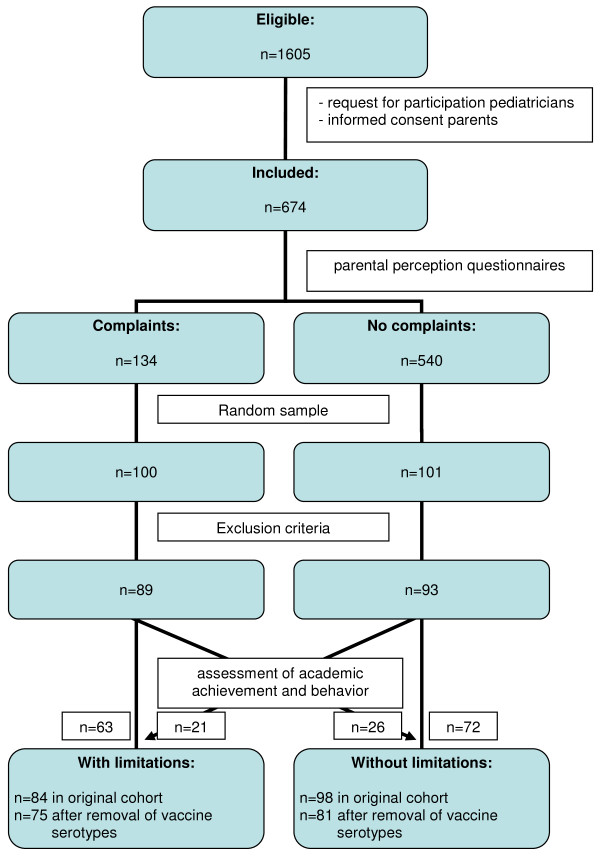
**patient flow chart for "academic or behavioral limitations"**.

Based on the two primary outcome measures "hearing loss" and "academic or behavioral limitations" and the risk factors found, the two prediction rules were developed using a multivariable logistic regression model. Potential risk factors were studied resulting, as mentioned in the introduction paragraph, in 5 independent risk factors in the hearing loss prediction model and in 9 factors in the prediction rule for academic or behavioral limitations [5,6]. Figure 1 and 2 show patient flow charts of the inclusion and assessment.

### Statistical analysis

We simulated the presumed effect of the vaccination by excluding all children who were infected by *S. pneumoniae *with one of the seven vaccine serotypes (4,6B,9V,14,18C,19F,23F) from both cohorts. Twenty six (9 with and 17 without limitations) of 182 children of the academic or behavioral limitations cohort and 70 (12 with and 58 without hearing loss) of 628 children from the hearing loss cohort were excluded. Two new prediction rules were calculated using this two adjusted cohorts.

Univariate logistic regression analysis was used to assess the effects of all original potential risk factors collected in the adjusted cohorts. Continuous variables were dichotomized when a linear relationship with the outcome could not be assumed. All variables with a p-value of less than 0.20 were included in a multivariate logistic regression model. This first selection step was needed because the number of potential risk factors compared to the group size was too large. The prediction models were developed using a forward selection method using a threshold p-value of 0.10 for variables to be included in the models. The area under the curve (AUC) of the receiver operating curve (ROC) was calculated to assess the discriminative ability of both prediction rules. A score for each risk factor was determined by dividing the (original, not rounded) regression coefficient of each variable in the model by the smallest regression coefficient of the model, multiplied by 10 and rounding off to the nearest integer. From each child a score was calculated by filling in the values from his or her risk factors in the prediction rules. These values are summarized in a table and a cut off score for having a high risk of developing hearing loss or academic or behavioral limitations was chosen to obtain the highest discriminative ability between groups. All analyses were carried out using SPSS statistical software edition 14.0 (SPSS inc, Chicago, US).

## Results

### Hearing loss

The regression coefficients, odds ratios and the contribution to the score of the five independent predictors of hearing loss after recovery from BM in childhood are presented in Table 1. These are the same five predictors that were also found in the previous prediction model [5], although the variable "duration of symptoms" has now been divided into three categories instead of two (namely < 1 day; 1-2 days, and > 2 days) because it was not significantly correlated with the development of hearing loss when divided in two categories (p = 0.12). *S. pneumoniae *still remained an important risk factor for children who were infected by a different serotype than the ones included in the pneumococcal vaccine. The regression coefficients and also the risk scores assigned to each variable were only slightly different from the previous prediction rule (AUC of the regression model after: 0.87 (95% confidence interval 0.82-0.93), versus 0.84 (95% confidence interval 0.78-0.91) in the original cohort) [5]. Table 2 shows a summary of the computed risk score for each child using the prediction rule.

**Table 1:** Independent predictors of hearing loss after bacterial meningitis.

Variable	Regression coefficient	Odds ratio (95% CI)	Contribution to risk score
Duration of symptoms			
< 1 day	RC		0
1-2 days	0.8	2.3 (0.7-7.2)	10
> 2 days	1.0	2.8 (1.0-8.0)	13
Absence of petechiae	2.4	11.0 (2.7-45.4)	29
CSF glucose level < 0.6 mmol/L	1.0	2.8(1.2-6.6)	13
*S. Pneumoniae*	1.1	3.2 (1.2-8.5)	14
(Transient) ataxia	3.3	28.4 (5.7-142.3)	40

Risk score = 10 × duration of symptoms 1-2 days + 13 × duration of symptoms > days + 29 × absence of petechiae + 13 × CSF glucose leven < 0.6 mmol/L + 14 × S. Pneumoniae + 40 × (transient) ataxia

AUC of the regression model:: 0.87 (95% confidence interval 0.82-0.93)

RC = reference category. For final risk score add all separate risk factors per child.

RC = reference category; CSF = Cerebrospinal fluid.

**Table 2:** Number of children with and without hearing loss across categories of the risk score. Values represent numbers (percentages).

Risk score	Children in the cohort n = 558(%), cumulative n(%)		Children with hearing loss n = 31 (%), cumulative n(%)		Children without hearing loss n = 527 (%), cumulative n(%)	
0-9	237 (42)		0 (0)		237 (45)	
10-23	82 (15)	319 (57)	2 (6)		80 (15)	317 (60)
24-39	84 (15)	403 (72)	4 (13)	6 (19)	80 (15)	397 (75)
40-56	140 (25)	543 (97)	16 (52)	22 (71)	124 (24)	521 (99)
≥57	15 (3)	558 (100)	9 (29)	31 (100)	6 (1)	527 (100)

**Figure 3:**
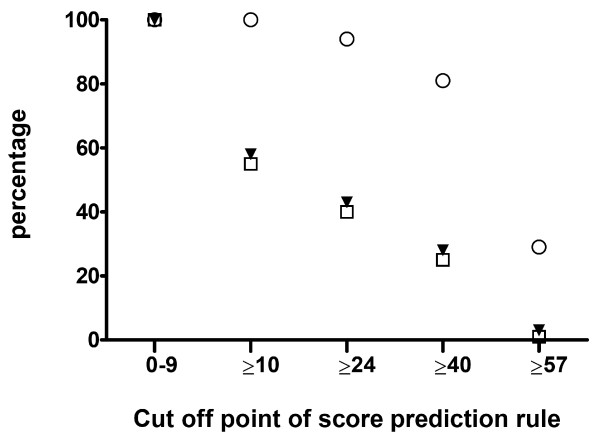
**positively vs falsely predicted as children with hearing loss using different cut off points**. **Legend: **▼ = % children in the total cohort (100% = 558 children). Ο = % positively predicted children with hearing loss (100% = 31 children). □ = % falsely predicted children without hearing loss (100% = 527 children)

When we applied the newly developed prediction rule on the post-pneumococcal vaccination cohort we could predict all children with hearing loss by using a cut off score of ≥ 10. No children with hearing loss had a score below 10; this means that a patient is at risk when one of the predictors is present. Three hundred and twenty one (82+84+140+15) of the total cohort of 558 children (58%) had a risk score ≥ 10, and were selected as being at risk of developing hearing loss. In the previous prediction model of Koomen et al. this was 62%, thus both rules have equally strong abilities to screen for hearing loss. Using this cut off score of ≥ 10, 290 (80+80+124+6) of 527 children (55%) without hearing loss are falsely predicted as children with hearing loss. Figure 3 gives an overview of the percentages of children positively and falsely predicted as children with hearing loss using different cut off points.

### Academic or behavioral limitations

Table 3 presents the predictors which were independently associated with the risk of developing academic or behavioral limitations after recovery from BM in childhood. All nine risk factors used in the original prediction rule of Koomen et al. remained significant [6]. However, the most striking differences compared with the previous model were the regression coefficients of the risk factor "delay of more than 6 hours between admission and start of antibiotics" which changed from 0.9 to 2.4 and the regression coefficient of birth weight ≤ 3000 gram which changed from 0.8 to 1.5. In both the old and new prediction rule, low birth weight was associated with a higher risk of developing limitations. The AUC of our new regression model after was 0.84 (95% confidence interval 0.78-0.90) which is comparable with the AUC of the model of the original cohort of Koomen et. al. (0.83;95% confidence interval 0.77-0.89) [6].

**Table 3:** Independent predictors of academic or behavioral limitations after bacterial meningitis.

Variable	Regression coefficient	Odds ratio (95% CI)	Contribution to risk score
Male gender	1.7	5.8 (2.3-14.5)	38
Birth weight ≤3000 gram	1.5	4.5 (1.7-12.1)	33
Educational level father^a^			
Lower education	2.0	7.3 (2.1-25.3)	43
Middle education	1.7	5.3 (1.6-17.1)	36
Higher education	RC		0
*S. pneumoniae *as causative pathogen	1.9	6.9 (1.7-28.3)	42
CSF leukocyte count/10000 cells (/3 mm^3^)	-0.5	0.6 (0.4-0.9)	-10
Delay of more than 6 hours between admission and start of antibiotics	2.4	11.2 (1.6-76.4)	53
Dexamethasone			
≤ 2 days	1.0	2.8 (0.5-14.1)	22
> 2 days	-0.8	0.4 (0.2-1.2)	-19
no	RC		0
Seizures treated with anticonvulsive therapy	-2.9	0.1 (0.0-0.4)	-62
Prolonged fever (> 9 days)	3.6	37.8 (3.3-435.6)	79

Score = 38 × Male gender + 33 × Birth weight ≤ 3000 gram + 43 × lower educational level father + 36 × middle educational level father + 42 × *S. Pneumoniae *- 10 × CSF leukocyte count/10000 cells (/3 mm^3^) + 53 × delay of more than 6 hours between admission and start of antibiotics + 22 × Dexamethasone ≤ 2 day - 19 × Dexamethasone > 2 days - 62 × Seizures treated with anticonvulsive therapy + 79 × prolonged fever.

AUC of the regression model: 0.84 (95% confidence interval 0.78-0.90)

RC = reference category; CSF = Cerebrospinal fluid.

^a ^Lower education = lower administrative or technical education (or less), middle education = secondary education, higher education = college or university education.

Table 4 shows a summary of the computed risk score for each child using the prediction rule. To demonstrate how the prediction rule for academic or behavioral limitations can be used we here give an example of a male patient (risk score: +38), with a birth weight of 3500 gram (risk score:0), a father with middle education level (risk score: +36), with *S. pneumoniae *as causative pathogen (risk score: +42), and a leukocyte count of 20000/cells in the CSF (risk score: -20). He had a delay of 8 hours between admission and start of antibiotics (risk score: +53), and no dexamethasone was used (risk score: 0). He developed no seizures (risk score: 0), nor prolonged fever (risk score: 0). His total risk score is than 149 and thus he has a high risk of developing academic or behavioral limitations.

**Table 4:** Number of children in the cohort with and without academic or behavioral limitations across categories of the risk score. Values represent numbers (percentages).

Risk score	Children in the cohort n = 166(%), cumulative n(%)		Children with academic or behavioral limitations n = 75(%), cumulative n(%)		Children without academic or behavioral limitations n = 91(%), cumulative n(%)	
≤29	19 (11)		0 (0)		19 (21)	
> 29 & ≤64,75	44 (27)	63 (38)	10 (13)		34 (37)	53 (58)
> 64,75 &≤94,66	48 (29)	111 (67)	18 (24)	28 (37)	30 (33)	83 (91)
> 94,66 & ≤134,5	38 (23)	149 (90)	30 (40)	58 (77)	8 (9)	91 (100)
> 134,5	17 (10)	166 (100)	17 (23)	75 (100)	0 (0)	91 (100)

**Figure 4:**
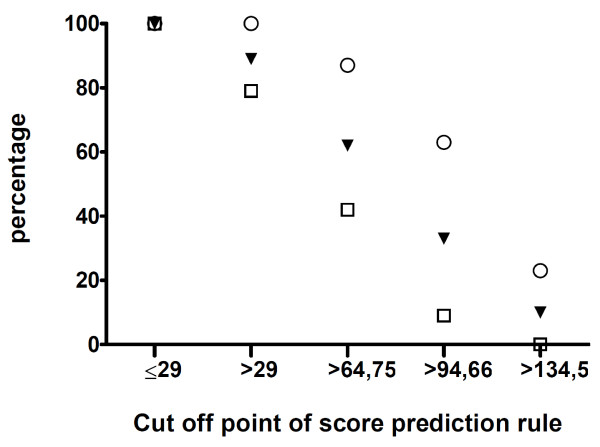
**positively vs falsely predicted as children with academic or behavioral limitations using different cut off points**. **Legend: **▼ = % children in the total cohort (100% = 166 children). Ο = % positively predicted children with academic or behavioral limitations (100% = 75 children). □ = % falsely predicted children without academic or behavioral limitations (100% = 91 children)

When we applied the newly developed prediction rule on the post-pneumococcal vaccination cohort, using a cut off score of > 64,75 we were able to positively predict 65 (18+30+17) of 75 children (87%) with limitations. This means that 103 (48+38+17) of the total of 166 children (62%) with a risk score > 64,75 are selected as possibly having limitations to reach 87% of positive predictions, whereas 38 (30+8+0) of 91 children (42%) without limitations are falsely predicted as children with limitations. With the previous prediction rule, 76% of the children with limitations could be positively predicted by selecting 38% of all children as having a high risk. So this new rule has less strong abilities to screen for limitations. Figure 4 gives an overview of the percentages of children positively and falsely predicted as children with limitations using different cut off points.

## Discussion

We studied the effect of the introduction of a seven valent conjugate pneumococcal vaccine on two previous developed prediction rules for hearing loss and academic or behavioral limitations in the Netherlands. The presumed effect of vaccination was simulated by excluding all children infected by *S. pneumoniae *with the serotypes included in the vaccine, from both previous collected cohorts. After calculating two new prediction rules we found that all original risk factors remained significant. So it can be assumed that the original prediction rules will also be applicable on a vaccinated population. The discriminative ability of both prediction rules remained as high as observed in the previous studies of Koomen et al. [5,6]. The risk factor "*S. pneumoniae *as causative pathogen" remained significant. This could be explained by the fact that the children infected by some of the non-vaccinated serotypes of this bacterium had a less favorable outcome than children infected by the PCV-7 serotypes or other pathogens [15], although there is not much evidence available for this phenomenon.

Some limitations of this study should be acknowledged. First item to address originates from the original prediction rules. From the cohort of 1605 children eligible, most children were excluded because the pediatricians or the parents did not want to participate. Although most of the basic patient characteristics as sex, age, and causative pathogen were very similar in both the original cohort and the included children, suggesting that these cohorts were representative for the original BM population, exclusion bias is possible. Further, the outcome measure "academic or behavioral limitations" was prospectively and extensively tested, but "hearing loss" was retrospectively assessed, leaving the possibility that some mild hearing problems were missed [5,6]. These limitations of the original studies also apply to the updated rules in this manuscript.

Second, the sample sizes included in the analyses are relatively small for developing new prediction models. General rules of thumb recommend at least ten cases per included variable. So the recommend number of children with hearing loss or limitations was 50 and 90 respectively, whereas our cohorts included 31 and 75 children with hearing loss or limitations, respectively. The variable ataxia included in the rule for predicting hearing loss and the variable prolonged fever in the rule for limitations had a very high Odds Ratio and a very large confidence interval. This may reflect this power problem. As mentioned before, the regression coefficient of the variable "delay of more than 6 hours between admission and start of antibiotics" changed from 0.9 tot 2.4, with also relatively high Odds Ratio and a large confidence interval. This striking difference may also be a result from low power.

The next issue concerns the applicability of this new constructed rule in clinical practice. Although we believe that the methodology and statistical analyses used are adequate, we do realize that the original rules are not externally validated yet. It is known that prediction rules might act significantly different when applied to a new cohort, making external validation and, if necessary updating, essential [16-19]. External validation of the original models is performed, but to come to an optimal result for the new situation, a cohort with vaccinated children should be constructed. But, at this moment the number of vaccinated children in the Netherlands is relatively small and follow up time is short, making external validation a difficult procedure. Further, the acceptable proportion of children with problems that were not predicted is disputable, making it difficult to recommend cut off points of the scores in clinical practice. Regarding hearing loss, we believe it is justifiable to say that hearing loss may not be missed at all, proposing a cut off score of ≥10. Regarding academic and behavioral limitations, the proportion of positively predicted children is 87% using a cut off point of 64,75, decreasing to 63% with a higher cut off point (figure 4). We believe this is a reasonable result, but practitioners may find this score to liberal.

Final point of discussion is the fact that the vaccine does not provide full coverage, although rates differ between countries and age-groups [8,9,20,21], and that serotype replacement is likely. This issue and an increase of invasive infections with non-vaccine serotypes has been observed in other countries [9-12,22]. The actual effect of vaccination and the amount of serotype replacement are unpredictable and it can take many years before a new balance in serotype prevalence is established [12,23]. Because BM has most impact in young children, especially those under the age of 1 year, vaccination is most relevant for this age group. It is therefore especially important to validate and update the prediction rules in this age group in future studies [16,19]. When validated, effort should be shifted to determine how to implement the clinical use of the prediction rules and thereby improve clinically outcome for BM surviving children.

## Conclusions

Although incidence is decreasing BM is still a serious infectious disease with high mortality and morbidity. The previously developed prediction rules showed that *S. pneumoniae *as causative pathogen is an important risk factor for sequelae. This study concludes that it can be assumed that the original prediction rules will also be applicable on a PCV-7 vaccinated population. However, the vaccine does not provide 100% coverage and there is evidence that serotype replacement will occur. The prediction rules need to be validated externally and further research is needed to investigate the impact of vaccination on serotype replacement.

## Competing interests

The authors declare that they have no competing interests.

## Authors' contributions

AEC, RdJ and CBT had primary responsibility for protocol development, data analysis and writing of the manuscript. IK, LS and AvF supervised the design and of the study and data analysis, and contributed to the writing of the manuscript. All authors read and approved the final manuscript.

## Pre-publication history

The pre-publication history for this paper can be accessed here:

http://www.biomedcentral.com/1471-2334/10/259/prepub
